# Peripartum cardiomyopathy: alluring challenge - case series and review of literature

**DOI:** 10.11604/pamj.2021.40.119.29168

**Published:** 2021-10-25

**Authors:** Youssra Bouhaddoune, Anas Hbali, Hanane Aissaoui, Asmae Mrabet, Nabila Ismaili, Noha El Ouafi

**Affiliations:** 1 Department of Cardiology, Mohammed VI University Hospital, Faculty of Medicine and Pharmacy of Oujda, Mohammed First University, Oujda, Morocco,; 2 Epidemiological Laboratory of Clinical Research and Public Health, Faculty of Medicine and Pharmacy of Oujda, Mohammed First University, Oujda, Morocco

**Keywords:** Dilated cardiomyopathy, peripartum cardiomyopathy, heart failure, pregnancy

## Abstract

Peripartum cardiomyopathy (PPCM) is a rare disease responsible for heart failure that usually occurs in the last month of pregnancy or within five months postpartum, without any other known cause. A case series of five PPCM patients admitted at the Department of Cardiology of the University Hospital Mohammed VI of Oujda, Morocco, between 2017 and 2019. All cases were represented by young (case 1: 35-year-old; case 2: 28-year-old; case 3: 30-year-old; case 4: 36-year-old; case 5: 34-year-old). All patients were multiparous who were admitted to our department with a severely reduced left ventricular ejection fraction. Case 1 and 4 were admitted 3 days after delivery for heart failure. Case 2 was admitted for cardiogenic shock after 3 months of delivery. Case 3 was admitted twelve days after delivery for acute heart failure with pulmonary embolism and multiple venous thrombosis. Case 5 had a history of PPCM was admitted for cardiogenic shock with a course marked by recurrent thromboembolic events. Case 1 and 2 responded to treatment at an early stage, case 4 has evolved to chronicity, the third patient died from an unclear cause, and the fifth patient died from a contraindicated pregnancy leading to the recurrence of fatal thromboembolic events. Above reported cases confirming the great heterogeneity in clinical presentation and course of peripartum cardiomyopathy and seems to confirm that a delayed diagnosis, as well thromboembolic complications are bad prognosis factors of these patients. Early diagnosis, multidisciplinary collaboration, prompt treatment of heart failure and continued monitoring are the keys to improve maternal survival.

## Introduction

Peripartum cardiomyopathy is a rare cause of dilated cardiomyopathy, it is manifested by congestive heart failure, due to left ventricular systolic dysfunction, that usually occurs in the last month of healthy women´s pregnancy or within five months postpartum [1]. Indeed, its etiology and clinical management, as well as its prognosis, are remains unknown. The incidence of peripartum cardiomyopathy varies between 1/3000 to 1/15000 pregnancies [2]. Peripartum cardiomyopathy represents 1% of the possible cardiovascular complications of pregnancy. Because of its low incidence and nonspecific symptoms, peripartum cardiomyopathy is often undetected or misdiagnosed. The diagnosis can be delayed, if symptoms are considered as a normal manifestation of pregnancy. When these symptoms are not diagnosed early, it leads to high mortality.

A case series of five PPCM patients with an objective to increase awareness of the disease in our region and help clinicians diagnose and plan their resources well. Early diagnosis and prompt treatment of heart failure could reduce peripartum cardiomyopathy complications and could restore the cardiac function in order to a good quality of life. Their clinical presentations were different and represented an important diagnosis challenge. The purpose of our study is to report 5 different cases and their management.

## Methods

**Setting**: this study was carried at the Department of Cardiology at the Mohammed VI University Hospital in Oujda (Morocco), between January 2017 to July 2019. The Mohammed VI University Hospital of Oujda, established in 2014, is a tertiary referral hospital for the oriental region of North-Eastern Morocco, with a capacity of 673 bed, who has specialty hospital, a mother-and-child hospital, a burn center, a legal medicine department, a central laboratory, emergency services, educational units, a boarding school and other administrative and technical premises. The Mohammed VI University Hospital also houses the Hassan II Cancer Center and the hospital for mental health and psychiatric diseases.

**Study population:** a critical patient's selection was used. The inclusion criteria in the study of hospitalized patients corresponded for a first group to the definition of the PPCM [3]: congestive heart failure secondary to left ventricular dysfunction that occurs in previously healthy women during the last month of pregnancy or during the five months after delivery with echocardiographic left ventricular dysfunction (EF of <45%) and/or left ventricular end-diastolic diameter of >2.7cm/m^2^ and without any other identifiable etiology. Our exclusion criteria were parturients with a previous heart disease, pregnant women or women who delivered with a heart disease developed outside the standard time limits, parturients with cardiac disease admitted to the obstetric gynecology department and who were not transferred to the cardiology department were also excluded. Epidemiological, clinical, paraclinical, therapeutic and follow-up data were retrospectively collected from patient´s medical records.

**Design:** we conducted a retrospective review of five cases hospitalized for peripartum cardiomyopathy in the Department of Cardiology of the University Hospital Mohammed VI of Oujda, over a period of 2 years, between January 2017 to July 2019. Clinical characteristics, management, and outcomes of patients are summarized in Table 1. Reporting of the current study is in accordance with the STROBE (Strengthening the Reporting of Observational Studies in Epidemiology) checklist [4].

**Table 1:** summary table of the clinical, echocardiographic characteristics, and outcomes of the study population

Case	1	2	3	4	5
Age	35	28	30	36	34
Parity, number	2	3	3	4	5
Comorbidities	Absent	Absent	Absent	Absent	Absent
Diagnosis, days PP	3	90	12	3	30
Symptoms	Dyspnoea; generalized edema	Dyspnoea; palpitation; lower-limb edema	Dyspnoea	Dyspnoea; palpitation; lower-limb edema	Dyspnoea
NYHA class	IV	III	IV	IV	IV
LVEF, %	21	17	20	22	20
LVEDD, mm	61	61	58	59	60
Complications	Heart failure	Cardiogenic shock; distal necrosis of the lower limbs; heart failure	Pulmonary embolism; pulmonary edema; multiple venous thrombosis; heart failure	Heart failure; pulmonary edema	Pulmonary embolism; cardiogenic shock; multiple venous thrombosis; atrial fibrillation; superior vena cava syndrome; heart failure
Evolution	Favorable	Favorable	Death	Favorable	Death

PP: peripartum; NYHA: New York Heart Association; LVEDD: left ventricular end-diastolic diameter; LVEF: left ventricular ejection fraction

**Data collection:** the collection of anamnestic, clinical, paraclinical, therapeutic, evolutionary and follow-up data for each patient was carried out from electronic medical records. Clinical data, including age, medical history, parity, day´s peripartum, presenting symptoms and identifiable risk factors were collected. ECG, cardiac enzymes, renal and liver function tests, thyroid profile and viral serologies were analyzed. Echocardiography parameters measured were left ventricular end diastolic dimension and left ventricular ejection fraction.

**Ethical considerations:** we obtained consent from the hospital head of clinical services to publish the data and have maintained patient anonymity.

## Results

The mean age was 32.6 years with a range of 28-36 years. All patients were multiparous and were diagnosed three days to three months after delivery. They had several previous pregnancies without PPCM, except one patient had a recurrence of PPCM after her fifth pregnancy. Cases were admitted to our department with a severely reduced left ventricular ejection fraction. The following is the detailed description of each case.

**Figure 1:**
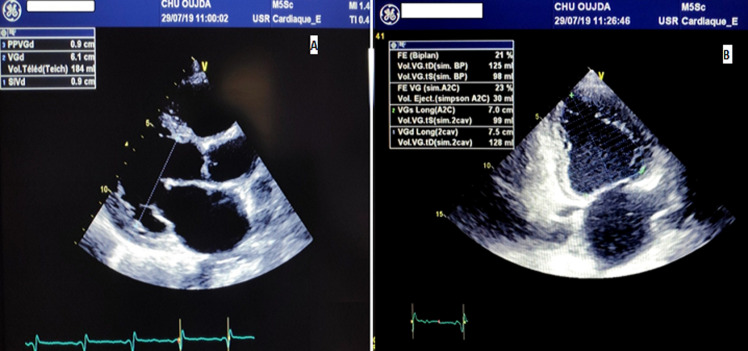
A) transthoracic echocardiography (TTE) in long-axis view showing dilated LF with LVEDD of 61 mm; B) apical 2-chamber, 2-dimensional echocardiogram showing severe systolic dysfunction at 21%

**Figure 2:**
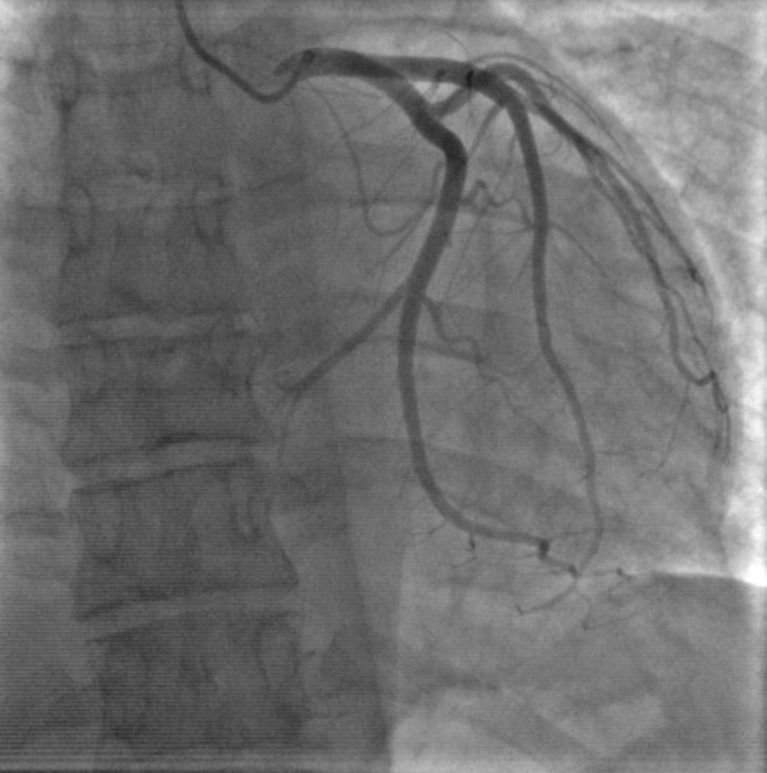
coronary angiogram showed normal coronary arteries

**Case 1:** a 35-year-old woman, multiparous (G2P2); without medical previous cardiac disease. The patient had an uneventful pregnancy, three days after vaginal delivery; she developed progressive dyspnea, orthopnoea and generalized edema. The clinical examination revealed signs of heart failure. ECG on admission showed sinus tachycardia, ventricular extrasystoles, and inverted T waves in precordial leads. Echocardiography showed dilated cardiomyopathy with left ventricular end diastolic dimension of 61 mm, global hypokinesia with severe left ventricular systolic dysfunction with ejection fraction of 21%, elevated filling pressure of left ventricular and mild mitral insufficiency; no left ventricle thrombus was noted (Figure 1A, B). The coronary angiogram revealed normal coronary arteries (Figure 2). Laboratory investigations revealed anemia (10.3 g/dl), the renal function test and troponin were normal. The thyroid hormone profile was normal, viral serologies (HIV/hepatitis B, C/syphilis) were negative. Our patient received injectable diuretics, angiotensin-converting enzyme inhibitor and low-dose of beta-blockers. She was discharged home 8 days after admission. After three months, the patient remains asymptomatic, echocardiography found left ventricular ejection fraction (LVEF) 55%.

**Figure 3:**
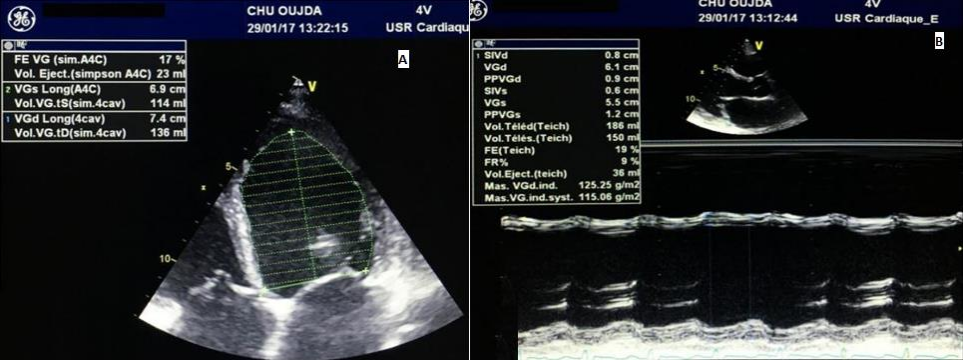
A) transthoracic echocardiography (TTE) in apical 4-chamber, 2-dimensional echocardiogram showing severe systolic dysfunction at 17%; B): M-mode transthoracic echocardiography on long-axis view showing dilated LF with LVEDD of 61 mm and severe systolic dysfunction

**Figure 4:**
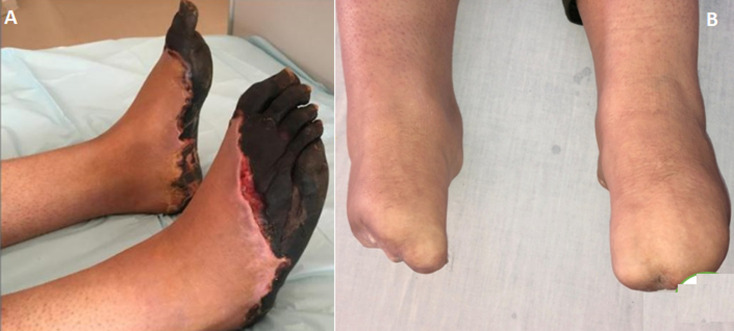
A) wound at the dorsal surface and the sole of the right foot; B): spontaneous loss of the toes of both feet

**Case 2:** a 28-year-old woman, multiparous (G3P3); without medical previous cardiac disease. After three months of vaginal delivery following normal pregnancy of her third child, she developed progressive dyspnea with important edema of the lower limbs and palpitation. She was admitted to our hospital with the signs of cardiogenic shock. At first examination, she was conscious, orthopnoea. She had a respiratory rate of 26 breaths/min and tachycardia at 150 beats/min, with a low blood pressure of 80/60 mmHg, with cold extremities. The rest of the examination revealed a jugular turgescence, hepatojugular reflux, and important lower-limb edema. Electrocardiography on admission revealed sinus tachycardia at 135 beats/min, with right bundle branch block and negative T-waves in precordial leads. Chest radiograph showed cardiomegaly with features of pulmonary venous hypertension. Laboratory investigations revealed anemia (hemoglobin: 9g/dl), infectious syndrome, impaired renal and liver functions. Transthoracic echocardiography revealed dilated cardiomyopathy with left ventricular end diastolic dimension of 61 mm, global hypokinesia with severe left ventricular systolic dysfunction with ejection fraction of 17%, elevated filling pressure of left ventricular, the cardiac index was 1.5 L/min/m^2^; no left ventricle thrombus was noted. At Doppler, there was minimal mitral leakage and tricuspid insufficiency (Figure 3A, B). She received inotropic drugs (dobutamine), diuretics, ivabradine, low molecular weight heparin and blood transfusion. After the resolution of cardiogenic shock, beta-blockers and spironolactone were started. After the regression of the lower limb edema, clinical examination showed bilateral toes necrosis (Figure 4A). Computed tomography angiography of the lower extremities showed no arterial abnormalities, it was secondary to low cardiac output. In the absence of viability, the amputation of the toes was indicated with debridement of necrotic wounds but refused by the patient. The evolution was marked by spontaneous loss of all feet toes (Figure 4B). Control echocardiography performed 6 months later, found an improvement of the left ventricular ejection fraction 56%.

**Case 3:** a 30-year-old woman, multiparous (G3P3); without medical previous cardiac disease, she was referred to our hospital, twelve days after vaginal delivery of her third healthy child, for acute heart failure with pulmonary embolism and multiple venous thrombosis. At first examination, she was conscious, orthopnoea. She had a pulse of 123 beats per min and blood pressure at 100/50 mmHg. She had signs of left pleural effusion, and lower limb edema with a negative Homan's sign. Electrocardiogram revealed sinus tachycardia. Laboratory investigations revealed anemia (hemoglobin: 10.1 g/dl), infectious syndrome, alteration of liver function tests, the renal function test and troponin were correct. The thyroid hormone profile was normal, viral serologies (HIV/hepatitis B, C/syphilis) were negative. Transthoracic echocardiography demonstrated a dilated left ventricle with severe left ventricular systolic dysfunction (ejection fraction=20%), and global left ventricular hypokinesia; no left ventricle (LV) thrombus was noted. There was mild mitral and tricuspid valve regurgitation. Computed tomography angiography showed bilateral pulmonary embolism with thrombosis extended to both jugular veins, subclavian veins and brachial veins. Venous Doppler ultrasound showed a mobile thrombus in the common femoral vein. The patient started an intravenous anticoagulation, as well as diuretics, spironolactone, angiotensin converting enzyme inhibitors and low-dose of beta-blockers. She was discharged home 1 month after admission on oral anticoagulation, without symptoms. One year later, the patient died at home, of an unexplained clear cause.

**Case 4:** a 36-year-old woman, multiparous (G4P4); without history or preexisting structural heart disease, she was admitted to our intensive care unit, with dyspnea at rest, palpitation and important edema of the lower limbs, three days after vaginal delivery of her fourth healthy child. The clinical examination revealed signs of heart failure. The electrocardiogram showed sinus tachycardia at 100 beats/min with secondary repolarization disorders. Chest radiograph showed cardiomegaly with pulmonary congestion. Transthoracic echocardiography showed dilated cardiomyopathy with left ventricular, global hypokinesia with severe left ventricular systolic dysfunction with ejection fraction of 22%; no left ventricle thrombus was noted. At Doppler, there was minimal mitral leakage and tricuspid insufficiency. Computed tomography (CT) pulmonary angiography was negative for pulmonary embolism. The thyroid hormone profile was normal, viral serologies (HIV/hepatitis B, C/syphilis, EBV) were negative. Our patient received injectable diuretics, angiotensin-converting enzyme inhibitors, spironolactone, and low-dose of beta-blockers. The patient was not adherent to her medication and remained symptomatic (dyspnea NYHA II). After 12 months, we noted a slight improvement of LVEF (from 22 to 35%).

**Figure 5:**
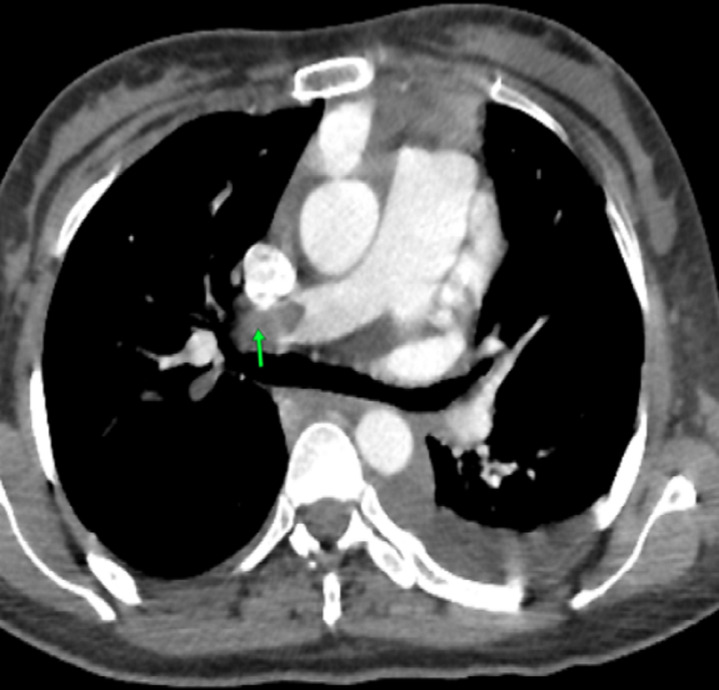
axial computed tomography pulmonary angiogram image showing pulmonary embolism of the right pulmonary artery

**Case 5:** a 34-year-old woman, multiparous, without cardiovascular risk factors. The patient had a history of peripartum cardiomyopathy, discovered three months after her fourth childbirth, with severe systolic dysfunction EF of 33%. She was put on medical treatment with a favorable evolution until the fifth unauthorized and unwanted pregnancy. One month after the cesarean section indicated for fetal distress, she was admitted to our hospital with the signs of cardiogenic shock. On examination, the woman was orthopnoea with signs of acute pulmonary oedema. She had a respiratory rate of 24 breaths/min and tachycardia at 100 beats/min with a low blood pressure of 80/60 mmHg, with cold extremities. The rest of the examination revealed a jugular turgescence, hepatojugular reflux and important lower-limb edema. Electrocardiography found passages in paroxysmal atrial fibrillation. CT pulmonary angiography found pulmonary embolism involving the right pulmonary artery (Figure 5). Venous Doppler ultrasound showed a mobile thrombus in the right common femoral vein. Transthoracic echocardiography demonstrated dilated cardiomyopathy with global hypokinesia and severe systolic dysfunction EF of 20%. Laboratory finding was anemia with hemoglobin at 10 g/dl, infectious syndrome (C-reactive protein (CRP) at 400 mg/l) whose starting point was lung infection. Prohormone brain natriuretic peptide (pro BNP) elevated to 8276 pg/ml, the renal function test, hepatic and troponin were normal. As part of the etiological assessment of dilated cardiomyopathy; thyroid hormones, viral serologies (HIV/hepatitis B, C/syphilis), vitamin dosage and antinuclear antibodies, were returning negative. After clinical improvement and shock resolution, she was put on diuretics, angiotensin-converting enzyme inhibitors, beta-blockers, antibiotics, spironolactone, amiodarone and curative anticoagulation base on acenocoumarol, she was discharged home 30 days after admission. After one month, she developed dyspnea at rest with generalized edema, and chest collateral circulation revealing a superior vena cava syndrome, confirmed on CT pulmonary angiography, showing extension of thrombosis to both internal jugular veins, subclavian veins, sigmoid sinus, brachial veins and superior vena cava, despite a concomitant acenocoumarol therapy (last INR at 2.8). We switched to low-molecular-weight heparins. Laboratory tests were performed in order to diagnose a thrombophilia, we found severe deficit in protein S (40% of activity) as well as low protein-C activity 37%. During hospitalization, the evolution was marked by a refractory heart failure with pulmonary aspergillosis, leading to patient death.

## Discussion

Peripartum cardiomyopathy is a rare cause of dilated cardiomyopathy, it is revealed by congestive heart failure, caused by left ventricular systolic dysfunction, that usually occurs in last month of healthy women pregnancy or within 5 months postpartum [1]. The incidence of peripartum cardiomyopathy varies between 1/3000 to 1/15000 pregnancies depending on the geographical regions, but it remains more frequent in Africa [2]. PPCM represents only 1% of the possible cardiovascular complications of pregnancy. Several risk factors are associated including age >30 years, black race, multiparity, pregnancy with multiple fetuses, obesity, arterial hypertension, preeclampsia, eclampsia and malnutrition [5]. In our series, four patients were >30 years, and all patients were multigravida.

The pathogenesis of peripartum cardiomyopathy remains unknown. It appears to be complex and multi-factorial. Several hypotheses have been proposed, but none of them could be confirmed [3]. These include viral myocarditis, auto-immune mechanisms, abnormal immune response to pregnancy, abnormal response to the hemodynamic stress of pregnancy, accelerated myocyte apoptosis, cytokine-induced inflammation, malnutrition, genetic factors, excessive prolactin production, abnormal hormonal function, increased adrenergic tone, and myocardial ischemia [6]. The diagnosis of peripartum cardiomyopathy can be made only after the exclusion of alternative etiologies of dilated cardiomyopathy [5]. The majority of women are diagnosed after childbirth, usually in the first month postpartum [7,8]. The diagnosis of PPCM is usually delayed, leading to development of preventable complications [7,9]. Most of the patients are presented with symptoms of heart failure [10,11]. A minority of patients will present thromboembolic complications, cardiogenic shock, and cardio-pulmonary arrest secondary to heart failure or arrhythmias [12]. The electrocardiogram may be normal or show sinus tachycardia with non-specific ST-segment or T-wave abnormalities [13], atrial fibrillation occurs occasionally in patients with PPCM [14]. Like all cardiomyopathies, transthoracic echocardiography is the key exam in peripartum cardiomyopathy. Echocardiographic criteria include systolic dysfunction with an ejection fraction <45% (and/or fractional shortening <30%) and left ventricular end-diastolic dimension (LVEDD) >2.7 cm/m^2^ [3,15]. Cardiac magnetic resonance imaging (MRI) has become a useful tool for the assessment of cardiac function and is increasingly being used to confirm and complement echocardiographic findings in PPCM patients [16]. In our series, all the patients were diagnosed three days to three months after delivery. All our patients developed symptoms of heart failure; dilated cardiomyopathy was found in all patients. The left ventricular systolic dysfunction was constant, the average left ventricular systolic function was 22.2% (range 17% -22%). The other abnormalities were: elevated left ventricular filling pressure (5 patients), the tricuspid (3 cases) and mitral regurgitation (5 cases), right ventricular (RV) dilatation in 4 cases, an alteration in the longitudinal systolic function of the RV in 3 cases.

Severe complications can be observed in the course of peripartum cardiomyopathy, including cardiogenic shock pulmonary edema, arrhythmias, thromboembolic events, distal necrosis of the lower limbs secondary to low cardiac output, and mortality [9]. All our patients, presented heart failure, two of them were complicated by cardiogenic shock, and three others had pulmonary edema, thromboembolic events including pulmonary embolism and multiple venous thrombosis were reported in two patients, atrial fibrillation and distal necrosis of the lower limbs were also observed in one case each.

In the absence of specific treatment, the treatment for peripartum cardiomyopathy is the same as for other forms of congestive heart failure. Patients with peripartum cardiomyopathy are at risk of developing severe thromboembolic events [17], to avoid these complications in women with peripartum cardiomyopathy and in those who have left ventricular ejection fraction <30%, anticoagulant therapy should be strongly considered [18]. A randomized study including 20 patients showed that long-term and short-term bromocriptine treatment, in addition to standard treatment of heart failure, could improve the functional prognosis of patients with peripartum cardiomyopathy and may improve heart function [8,19]. All our patients received medical treatment based on diuretics and oxygen therapy. The angiotensin-converting enzyme inhibitors and the beta-blockers were prescribed respectively to 3 and 4 patients, the use of dobutamine was requested in two patients that presented cardiogenic shock, amiodarone was prescribed in a single patient in atrial fibrillation, anticoagulation was indicated for 3 patients.

The mortality rate between 5% to 25% [20]. Complete recovery is possible in 23 to 41% of cases [6]. Pregnancy after diagnosis is not recommended, due to the risk of cardiac decompensation, which puts both mother and baby at mortality risk. In our study, follow up was favorable in two patients, with complete clinical regression of echocardiographic abnormalities. Case 1 and 2 responded to treatment at an early stage, case 4 has evolved to chronicity. Two deaths occurred, the third patient died from an unclear cause, one year after her hospitalization, and the fifth patient died from a contraindicated pregnancy leading to the recurrence of fatal thromboembolic events.

**Limitations:** this study was limited by its retrospective and the small sample size nature, and unavailability of follow-up data in a significant proportion of women. Future research with prospective enrollment and longer follow-up is needed.

## Conclusion

Peripartum cardiomyopathy is a rare pathology affecting healthy young women; the etiology remains unknown, mortality rated high. Early diagnosis, multidisciplinary collaboration, prompt treatment of heart failure and continued monitoring are the keys to improve maternal survival. Peripartum cardiomyopathy deserves further scientific attention, from critical-care medicine, cardiologists and general practitioners, to improve diagnostic and management.

### What is known about this topic

- Peripartum cardiomyopathy is a relatively rare pathology and the etiology remains unknown;
- The diagnosis of peripartum cardiomyopathy can be made only after the exclusion of alternative etiologies of dilated cardiomyopathy;
- The treatment is not specific, it is based on the treatment of congestive heart failure.

### What this study adds

- Thromboembolic events represent the number one cause of mortality;
- Physician should be vigilant to the first signs and symptoms of heart failure which may not be seen, they are often considered as abnormal part of pregnancy.
